# IL-36/IL-36R signaling promotes CD4^+^ T cell-dependent colitis via pro-inflammatory cytokine production

**DOI:** 10.3389/fimmu.2025.1604332

**Published:** 2025-06-26

**Authors:** Maya Maarouf, Michal Kuczma, Timothy L. Denning

**Affiliations:** Institute for Biomedical Sciences, Center for Inflammation, Immunity, and Infection, Georgia State University, Atlanta, GA, United States

**Keywords:** inflammatory bowel disease, T cell mediated colitis, adoptive transfer, IL-36G, IFNg signaling, Crohn’s disease, ulcerative colitis

## Abstract

**Background:**

Inflammatory bowel disease (IBD) is a multifactorial, chronic disease that affects approximately 1.5 million people in the United States. Several important factors are implicated in the pathogenesis of IBD, one factor being dysregulation of the immune system. This dysregulation results in the accumulation and stimulation of innate and adaptive immune cells, and subsequent release of soluble factors, including pro-inflammatory cytokines. One of these cytokines is a member of the IL-36 cytokine family, IL-36γ, which is overexpressed in human IBD and experimental models of colitis. In this study, we explored the role of IL-36γ in promoting CD4^+^ T cell activation and cytokine secretion.

**Methods:**

Spleens and lymph nodes were collected from wild-type and IFNγ^-/-^ mice and were processed into cell suspensions to isolate naïve CD4^+^ T cells. Naïve CD4^+^ T cells were stimulated with IL-36 cytokines. IFNγ and TNFα were evaluated by ELISA using cell culture supernatants, and cell culture pellets were used to isolate RNA for qPCR. Naïve CD4^+^ T cells previously stimulated in the presence or absence of IL-36γ, from wild type (WT) or IFNγ-/- mice were transferred to Rag-/- mice to induce colitis. Fecal pellets were collected from mice during disease to analyze lipocalin (LCN2) levels from fecal supernatants. After euthanasia, colons, spleens, and mesenteric lymph nodes were harvested and processed into cell suspensions for intracellular cytokine staining.

**Results:**

Our results demonstrate that IL-36γ stimulation of naive CD4^+^ T cells significantly induced IFNγ expression *in vitro* and was associated with augmented intestinal inflammation *in vivo* using the T cell transfer model of colitis. Using IFNγ^-/-^ naive CD4^+^ T cells, we observed a dramatic decrease in the ability of these cells to produce TNFα. Moreover, the transfer of these cells to Rag^-/-^ mice did not cause robust colitis.

**Conclusion:**

These data not only suggest that IL-36γ is a regulator of a pro-inflammatory cytokine network involving IFNγ and TNFα but also highlights the importance of targeting IL-36γ and IFNγ as therapeutic approaches. Our studies have broad implications in relation to targeting specific cytokines in human IBD.

## Introduction

Inflammatory bowel disease (IBD) is a multifactorial, chronic condition that remains incurable until this day, with its two main forms being Crohn’s disease (CD) and ulcerative colitis (UC). While CD affects any part of the gastrointestinal (GI) tract, UC impacts the colon only. Regardless, they are both characterized with intestinal inflammation and epithelial barrier injury (1). To date, the types of therapeutics currently available primarily work to reduce and suppress inflammation, without curing the disease (2). Unfortunately, the etiology of IBD remains ambiguous. Several factors play a role in the pathogenesis of IBD, including genetics, environmental factors, the microbiota, and immunological dysregulation (3, 4).

The intestinal epithelial barrier acts as a defense barrier between the intestinal mucosa and the lumen, composed of a layer of intestinal epithelial cells (IECs) (5). Disruption of the mucosal barrier increases intestinal permeability (6, 7), resulting in immune dysregulation with leukocyte infiltration. This triggers intestinal inflammation and a primary immune response in mesenteric lymph nodes, where naïve CD4^+^ T cells interact with the antigen to differentiate into activated effector cells. The effectors then polarize into any of the different T cell subsets, T helper cell type 1 (Th1), Th2, Th17, or regulatory T cells (Tregs), depending on the microbial challenge (8). Th1 cells, which are especially seen in CD patients and induced by IL-12, function by producing IFNγ and TNFα. On the other hand, UC is thought to have more of a Th2 mediated response, producing IL-4, IL-5, and IL-13. The Th17 subset, also found in IBD patients, is induced by IL-6 production and the cytokine TGFβ and plays a role in maintaining commensal microbiota (9). While the mentioned subsets are classified as pro-inflammatory, Tregs are known for their inhibitory effects and ability to dampen immune responses by producing anti-inflammatory cytokines like IL-10 and expressing the transcription factor Foxp3 (7). Overall, CD4^+^ T cells are strongly associated with IBD pathogenesis (10), and their cytokine production can cause an imbalance allowing for disease progression (11).

As mentioned previously, IFNγ is upregulated in CD and UC as well as experimental murine colitis (12) and is thought to be a major driver of the amplified immune response seen in intestinal inflammation. IFNγ is produced by several types of immune cells, one of them being T cells. An increased expression of several IFN-regulated genes was observed in many autoimmune diseases (13). In IBD and experimental Dextran Sulfate Sodium (DSS) colitis, IFNγ contributes to vascular barrier disruption, worsening colonic inflammation and increasing intestinal blood vessel permeability which contributes to structural and functional perturbations (14). Due to the barrier disruption, more luminal antigens are translocated to the lamina propria; thus, exacerbating the immune response. Studies that used endothelial cell-specific IFNγ receptor knockout mice with induced DSS colitis showed decreased signs of disease inflammation (14). Targeting IFNγ signaling has been proposed as a potential therapeutic strategy for many autoimmune diseases, though its role in IBD needs further investigation.

In addition to IFNγ, the IL-1 superfamily of cytokines is also involved in the pathogenesis of IBD. This superfamily consists of the following cytokines: IL-1α, IL-1β, IL-18, IL-33, and IL-36. Previous studies have explored blocking IL-1 signaling by using Anakinra, a recombinant form of the IL-1 receptor antagonist (IL-1Ra) to inhibit IL-1β activity. Data revealed that Anakinra can alleviate experimental colitis in Winnie-TNF-KO mice. Canakinumab, which targets IL-1β and used to treat several autoimmune diseases, has been found to be effective in treating early onset IBD or older pediatric and adult IBD patients (15). Given that IL-18 is found in elevated levels in UC patients, a study that used wild type (WT), IL-18^-/-^, and IL-18R^-/-^ mice in DSS colitis model found that IFNγ production and IL-17 producing CD4^+^ T cells were reduced in the colons of both knockout models, along with less severe colitis than WT. Treating these mice with oral IL-18 inhibitor decreased Th1 cells and neutrophils, as well as disease severity (16). Another study tested the efficacy of a monoclonal anti-mature IL-18 antibody in DSS colitis mice found that it improved acute and chronic DSS colitis, reduced production of IFNγ and TNFα and ameliorated the epithelial cell barrier (17). When tested in combination with anti-TNFα, this regimen significantly reduced body weight loss, ameliorated intestinal inflammation, and decreased leukocyte infiltration, overall improving colitis (17). IL-33 is also known for its role in intestinal homeostasis, along with its receptor ST2, where their dysregulation has been shown to play a role in IBD pathogenesis, specifically in tissue damage (18). Studies suggest that IL-33 may have more of a protective role in experimental colitis; IL-33 deficient mice were found to be highly susceptible to colitis. In the 2,4,6-trinitrobenzene sulfonic acid (TNBS) colitis model, some studies found that the administration of recombinant IL-33 ameliorated colitis symptoms, while others showed it attenuated colitis via a Treg driven response. However, IL-33 was also found to play a varying role depending on the stage of the disease (19).

Finally, the role of the IL-36 cytokine family has been far less explored. This family of cytokines includes three agonists: IL-36α, IL-36β, and IL-36γ. These ligands and their receptor, IL-36R, are expressed mostly on epithelial cells, and some of their target cells include IECs and naïve CD4^+^ T cells (20, 21). Binding to IL-36R activates nuclear factor kappa B (NF-κB) and mitogen-activated protein kinases (MAPK) (21), causing inflammation. However, IL-36 cytokines are regulated by their natural inhibitor, IL-36Ra, which inhibits the inflammatory signaling pathway by competing with the IL-36R ligands and inhibiting NFκB activation (22). IL-36α and IL-36γ, specifically, function at barrier tissues, such as the intestines and are pro-inflammatory in IBD. Additionally, IL-36α and IL-36γ levels have been found to be elevated in IBD patients and are expressed by IECs, lymphocytes, and macrophages. IL-36 signaling thus promotes inflammation, making IL-36/IL-36R a potential therapeutic target for IBD. Spesolimab, a humanized monoclonal antibody that targets the IL-36 pathway is currently FDA approved for the treatment of autoimmune diseases, such as generalized pustular psoriasis, and has been tested in patients with moderate to severe UC in phase 2 studies to evaluate its safety, efficacy, and mechanism of action. While it was generally well-tolerated by patients, efficacy endpoints were not met (23).

Although significant progress has been made towards managing IBD, understanding the role of IL-36 cytokine family in IBD pathogenesis is crucial since the antagonist IL-36γ is elevated in human IBD and experimental colitis, as well as other inflammatory diseases. While anti-TNFα therapy can induce clinical remission in CD patients (24), the disease ends up relapsing in more than 60% of patients (25, 26). Cytokine blockers and immunomodulators have recently been developed to treat intestinal inflammation. However, patients can still go into clinical relapse. Therefore, evaluation of IL-36/IL-36R signaling in T cell-mediated colitis will further narrow down therapeutic targets for intestinal inflammation. Further, the IL-36/IFNγ pathway has not been assessed in the adoptive transfer model of colitis.

## Materials and methods

### Mice

The following mice were obtained from the Jackson Laboratory: Wild-type C57BL/6J (B6 WT), B6.129S7-*Rag1^tm1Mom^*/J (Rag^-/-^), and B6.129S7-*Ifng^tm1Ts^*/J (IFNγ^-/-^). Breeder B6 and Rag^-/-^ mice were purchased and subsequently bred at Georgia State University Division of Animal Resources. All animal procedures were performed according to the Guide for the Care of Use of Laboratory Animals, under *institutional animal care and use committee* (IACUC) guidelines. All mice involved in experimental procedures were eight to twelve weeks old, both females and males.

### Cell culture and supernatant harvesting for ELISA

Spleens and lymph nodes were collected from eight-week-old WT and IFNγ^-/-^ mice, both females and males. These organs were processed into cell suspensions. Splenocytes were resuspended in Ammonium-Chloride-Potassium (ACK) buffer for five minutes to lyse erythrocytes then washed once with PBS. Cells from splenocytes and lymph nodes were combined together to isolate naïve CD4^+^ T cells via EasySep™ Mouse Naïve CD4^+^ T Cell Isolation Kit (Stemcell). The enriched cells were checked for purity by staining for CD4, CD45RB, and CD25. The desired population was CD4^+^ CD45RB^hi^ CD25^-^. These cells were resuspended in complete medium.

For cell culture, 96-well flat bottom plates were coated with 5 μg/mL αCD3 (BioLegend) and 1 μg/mL αCD28 (BioLegend) and left overnight at 4°C for maximum binding. The plate was washed three times with PBS the next day. Naïve CD4^+^ T cells were plated at 200,000 cells per well, in the presence or absence of IL-36α, IL-36β, and IL-36γ (R&D Systems) at a concentration of 100 ng/mL. In the latter experiments, IFNγ neutralizing antibody (referred to as αIFNγ; clone XMG.1; Thermofisher) was used at a concentration of 2 μg/mL. The plate was then placed in the incubator for 48 hours at 37°C.

To harvest supernatant, the 96-well culture plate was spun down at 500 x g for 5 minutes at 4°C. The supernatant was aspirated into 1.5 mL Eppendorf tubes and stored at -80°C for long-term use. IFNγ and TNFα ELISA kits were obtained from Invitrogen and performed according to the manufacturer’s protocol.

### Adoptive T cell transfer colitis model

Splenocytes and lymph node cells were harvested from eight-week-old WT and IFNγ^-/-^ mice, both females and males. Cells were processed and enriched for naïve CD4^+^ T cell (CD4^+^ CD45RB^hi^ CD25^-^), as described above.

After enrichment/sorting, cells were checked for purity. This was done by staining for CD4, CD45RB, and CD25. The desired population was CD4^+^ CD45RB^hi^ CD25^-^. Cells were later resuspended in phosphate buffered saline (PBS) at a concentration of 2.5 x 10^6^ cells/mL. 500,000 cells were transferred into each Rag^-/-^ mouse via intraperitoneal (IP) injection. Colitis then developed between ten to twelve weeks. Activity, body weight, and stool consistency were recorded weekly for all mice, until euthanasia. Activity was assessed and measured based on changes in behavior, physical activity (e.g lethargy, running around cage, and resistance when trying to scruff), and time in active exploration and social interactions. Stool consistency was based on the physical stool texture, specifically solid pellets, loose/soft stool, or watery diarrhea.

### Hematoxylin and eosin staining and histological score evaluation

At the end of the experiment, colons were harvested from all mice. After removal of fecal matter, pieces of colonic tissue were fixed in 10% formalin at room temperature. Paraffin embedding, sectioning, and H&E staining was performed by HistoWiz. An individual histological score (ranging from 0 to 4) was attributed for degree of inflammation and immune cell infiltration using a scale of colonic inflammation (27) as a result of cytokine imbalance, as such 0= no infiltrate/inflammation, 1=minimal mucosal infiltrate and minimal hyperplasia, 2= mild mucosal infiltrate with mild hyperplasia and some erosions, 3= moderate mucosal and submucosal infiltrate with moderate hyperplasia and goblet cells loss with some crypt abscesses, 4= marked mucosal and submucosal infiltrate with marked hyperplasia and multiple crypt abscesses.

### Quantification of fecal lipocalin (LCN2) by ELISA

Fecal pellets were collected from mice during the course of disease and frozen at -80°C. To harvest fecal supernatants, the frozen fecal samples were resuspended in PBS at a concentration of 100 mg/mL and left at 4°C overnight to allow the feces to soften. The next day these samples were homogenized for 1 minute and then centrifuged at maximum speed for 15 minutes at 4°C. The supernatant was aliquoted and frozen at -80°C.

To analyze LCN2 levels, Mouse Lipocalin-2/NGAL DuoSet ELISA kit by R&D Systems was used and followed according to the manufacturer’s protocol. Fecal supernatants were diluted with reagent diluent to fit under the standard curve.

### Intracellular cytokine staining

At the end of the study, colons, spleens, and mesenteric lymph nodes were harvested. To process colon tissue, the tissue was cut for easier cleaning of the feces. Tissue was continuously washed in PBS. The colon tissue was pre-digested in HBSS and 2 mM of EDTA at 37°C by shaking for 15 minutes at 150 x g. Then, the colon was cleaned of mucus using a tissue paper and flushed again with PBS. A second HBSS and 2 mM EDTA shaking step was performed as described above. After cleaning the tissue of mucus and flushing with PBS, enzymatic digestion of tissue pieces was done with 1 mg/ml of collagenase type IV and 40 μg/ml of DNAse I and shaking for 30 minutes at 150 x g. The cell suspension was then centrifuged and filtered by running the cell suspension through glass wool.

Spleens were processed as mentioned previously and by lysing erythrocytes with ACK buffer for 5 minutes and washing with PBS. Mesenteric lymph nodes were processed the same way, without the need to lyse for erythrocytes.

The final cell suspensions were resuspended in complete medium. Desired cells were plated in a tissue culture plate to be stimulated for cytokine production. 50 ng/mL phorbol myristate acetate (PMA), 1 μg/mL ionomycin, 1:1000 Brefeldin A, and 1:1000 monensin were added to each well and placed in the incubator at 37°C for 4 hours. The cells were then centrifuged and stained with 1:1000 Live/Dead dye for 5 minutes at room temperature. After a PBS wash, the cells were stained for CD4, CD44, and Ly6G for 20 minutes in the dark. The cells were centrifuged again and washed with PBS, then fixed by resuspending the cell in Fixation/Permeabilization Solution for 20–30 minutes in the dark at room temperature. The cells were centrifuged and washed in PBS, then stained for cytokines for 20–30 minutes in the dark at room temperature. TNFα fluorochrome antibodies were diluted in Permeabilization buffer. The cells were later washed with PBS once and resuspended again in PBS for flow cytometry. Compensation was performed using single-stained cells to account for fluorescence spillover between fluorophores. Additionally, unstimulated samples were included as negative and gating controls for measuring TFNα production. These data were analyzed on Flowjo.

### Statistical analysis

All statistical analyses were performed with GraphPad Prism software, version 9.0 (Graphpad Software). When 2 conditions were present, a t-test (parametric) was performed. If parametric one-way ANOVA for 3 or more groups was performed, normality assumption was met. One-way ANOVA and Tukey’s Multiple Comparison Test or Student’s t test were used to determine significance. *p < 0.05, **p < 0.01, ***p < 0.001; ****p <0.0001; n.s.= not significant.

## Results

### IL-36*γ* increases naïve CD4^+^ T cell proliferation, clustering, and IFN*γ* production *in vitro*

**Figure 1:**
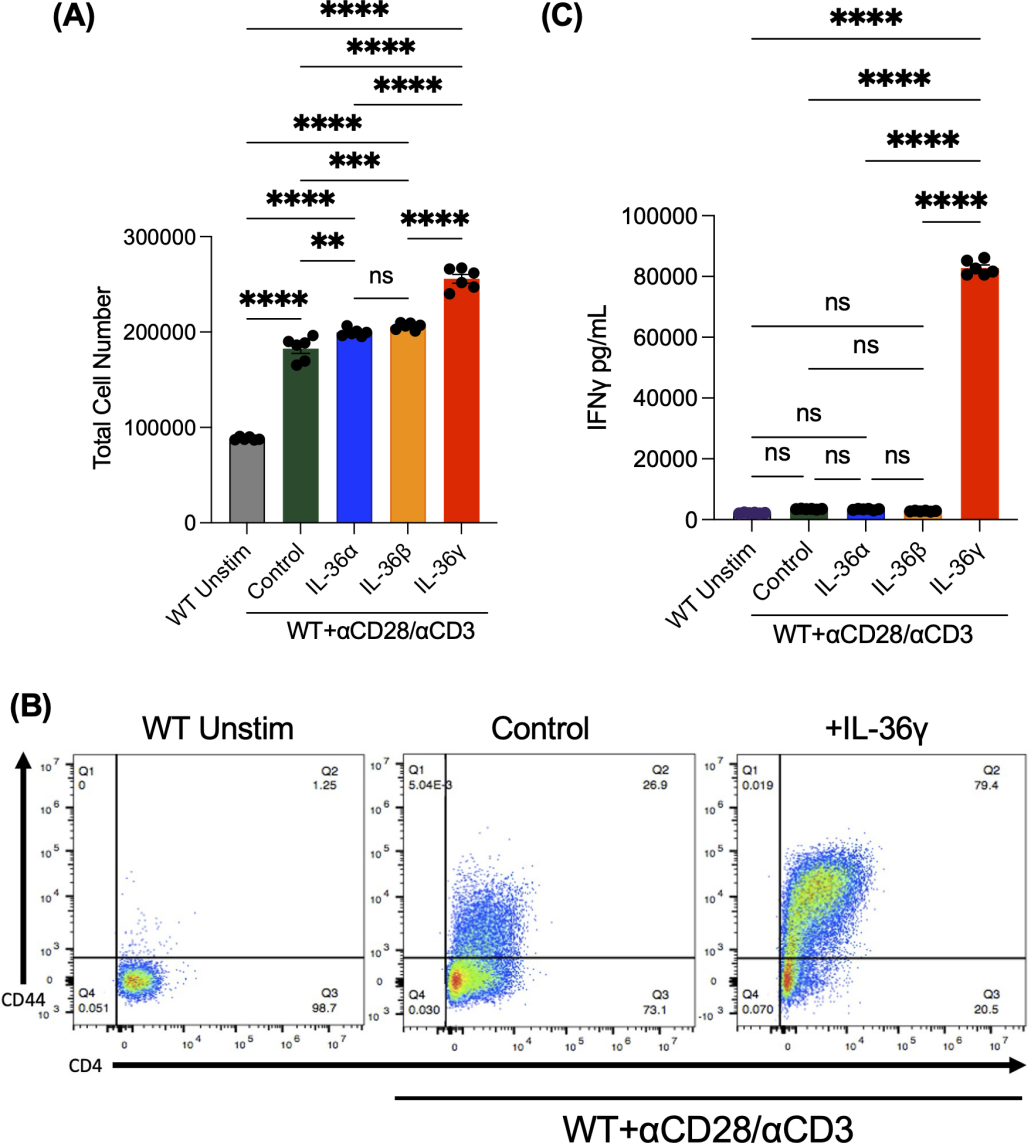
IL-36γ increases naïve CD4+ T cell proliferation, clustering, and IFNγ production *In Vitro*. Naive CD4+ T cells were isolated from splenocytes and were stimulated on a pre-coated plate with αCD3/αCD28 in the presence of IL-36α, IL-36β, or IL-36γ. Some cells were left unstimulated as a control. These data were collected at 48 hours. **(A)** Cells were counted under a hemocytometer using Trypan Blue. **(B)** The expression of the activation marker CD44 was evaluated after 48 hours, which was found to be highly expressed on cells stimulated with IL-36γ. **(C)** Supernatants from these cultures were checked for the pro-inflammatory cytokine IFNγ by ELISA. All data are presented as mean ± SEM; p < 0.05, **p < 0.01, ***p < 0.001; ****p <0.0001; n.s., not significant, one-way ANOVA with Tukey’s multiple comparison test.

Previous studies in mice have shown that IL-36β can increase naïve CD4^+^ T cell proliferation and induce pro-inflammatory cytokine production and Th1 polarization (22). Given this, we investigated the influence of the three IL-36 ligands, IL-36α, IL-36β, and IL-36γ, on naïve CD4^+^ T cells. To do so, splenocytes were harvested from WT B6 mice, and naïve CD4^+^ T cells were isolated. These cells were stimulated with αCD3/αCD28 for 48 hours in the presence or absence of the IL-36 ligands. As a negative control, some cells were left unstimulated. Cells were enumerated 48 hours following culture and supernatant was collected to test for IFNγ production. We observed an increase in cell numbers that were cultured in the presence of IL-36 ligands, especially in cultures that were stimulated with IL-36γ (**Figure 1A**). As the same number of cells were plated in each group and no difference in cell death was observed, the increase in cell number in IL-36γ-stimulated cultures was likely not due to a survival effect (data not shown). To further assess cell activation, we checked for the activation marker CD44 by flow cytometry and observed a robust increase in CD44 expression on the highly activated IL-36γ-stimulated cells (**Figure 1B**). Moreover, cells stimulated in the presence of IL-36γ demonstrated a significant induction of the Th1 cytokine, IFNγ, (**Figure 1C**) compared to the control samples (cells stimulated with αCD3/αCD28).

### Transfer of IL-36*γ* stimulated naïve CD4^+^ T cells into Rag^-/-^ mice exacerbates T cell-mediated colitis

**Figure 2:**
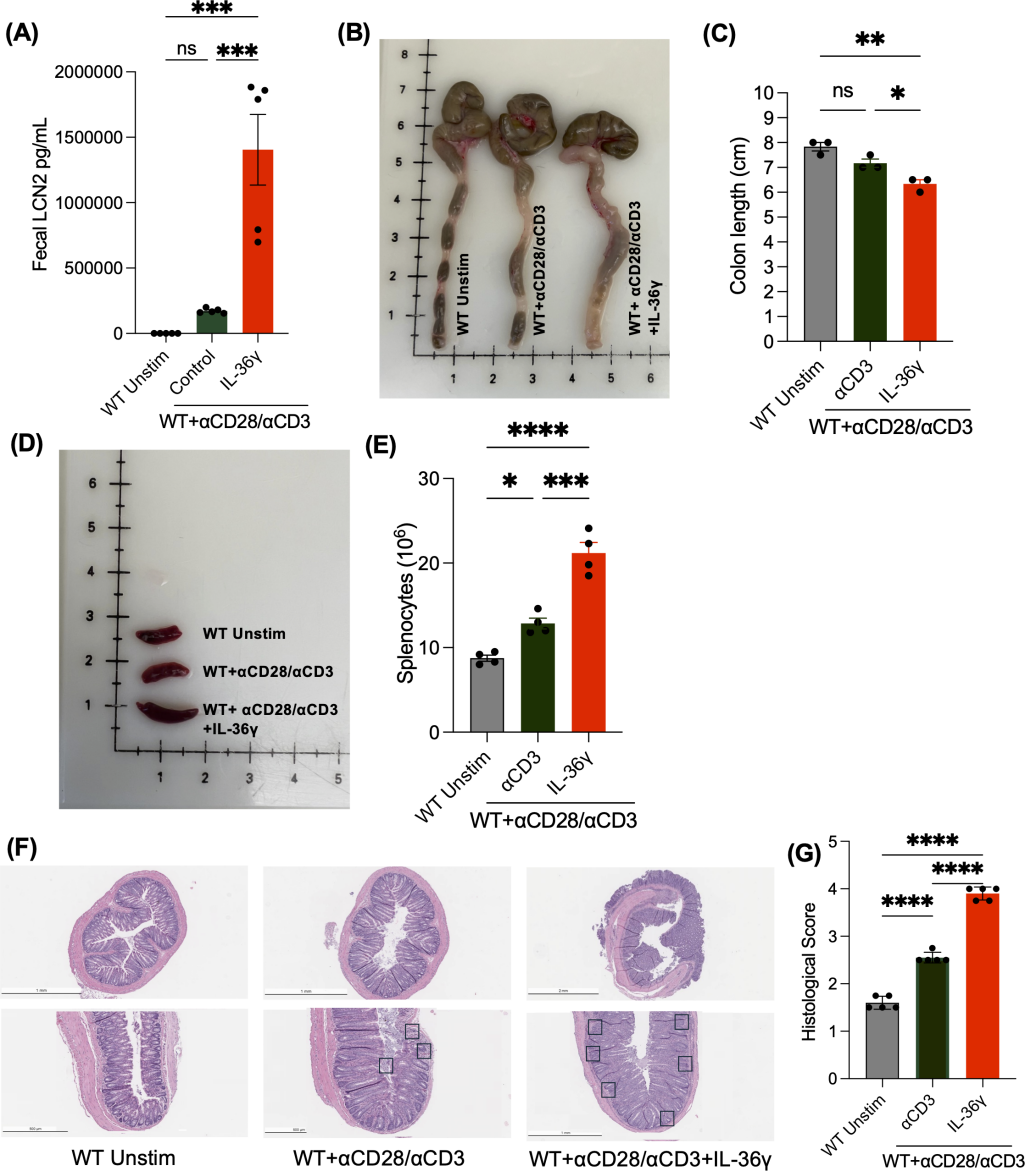
Transfer of IL-36*γ* stimulated naïve CD4^+^ T cells into rag^-/-^ mice exacerbates T cell-mediated colitis. **(A)** Lipocalin 2 (LCN2), a colitis inflammatory biomarker, was measured by enzyme-linked immunosorbent assay (ELISA) from fecal samples at time of sacrifice from mice that were adoptively transferred with unstimulated naïve CD4+ T cells or naïve CD4+ T cells stimulated with αCD3 +/- IL-36γ. **(B, C)** Images of colons and a comparison of their lengths, respectively, collected from the mice, upon euthanasia, show a shorter colon length, along with thickened tissue in mice that received naïve CD4+ T cells stimulated with IL-36γ. **(D, E)** Images of spleens after mice were euthanized at 10 weeks show splenomegaly and elevate splenocyte number in mice that received IL-36γ stimulated naive CD4^+^. **(F)** The H&E staining of colon sections from the mice groups mentioned is shown. **(G)** Histology scoring of colon sections from mice that were treated as mentioned. All data are presented as mean ± SEM; *p < 0.05, **p < 0.01, ***p < 0.001; ****p <0.0001; n.s., not significant, one-way ANOVA with Tukey’s multiple comparison test.

Next, we evaluated the impact of IL-36γ-induced IFNγ secretion using the T cell-mediated colitis model. CD4^+^ T cells activated in the presence or absence of IL-36γ were transferred into Rag^-/-^ mice. Unstimulated naïve CD4^+^ T cells were transferred into Rag^-/-^ mice as a control. Weight, activity, stool consistency, and fecal lipocalin (LCN2) levels were monitored over 10 weeks. Mice transferred with IL-36γ-activated CD4^+^ T cells appeared far less active than counterparts that received unstimulated cells. Recipients of IL-36γ-activated CD4^+^ T cells also exhibited softer stool consistency and secreted the highest levels of fecal LCN2 (**Figure 2A**). At 10 weeks post transfer, all mice were euthanized, and their spleens and colons were harvested for downstream analysis. Colons from the IL-36γ group were shorter in length than the other two groups and appeared more thickened and inflamed (**Figure 2B**). The group that received the WT unstimulated cells and anti-CD3/CD28 stimulated cells had colons that measured around 7.5–8 cm, while the colon of mice that received IL-36γ-stimulated CD4^+^ T cells measured around 6.5 cm (**Figure 2C**). Moreover, colons from the unstimulated group had well-formed solid fecal pellets, while the stool was of a much softer consistency in the other two groups, especially in the group that was treated with IL-36γ-stimulated CD4^+^ T cells (**Figure 2B**), which turned into diarrhea 7 weeks post transfer. The recipients of IL-36γ-stimulated CD4^+^ T cells also harbored a visibly enlarged spleen (**Figure 2D**). Corroborating this, the total number of splenocytes of recipients of the IL-36-stimulated CD4^+^ T cells were increased in comparison to the other groups (**Figure 2E**). Further, H&E staining revealed robust leukocyte infiltration in this group (**Figure 2F**), and therefore, the highest histological score (**Figure 2G**).

### IL-36g-induced TNF*α* production by naïve CD4^+^ T cells is IFNγ dependent

**Figure 3:**
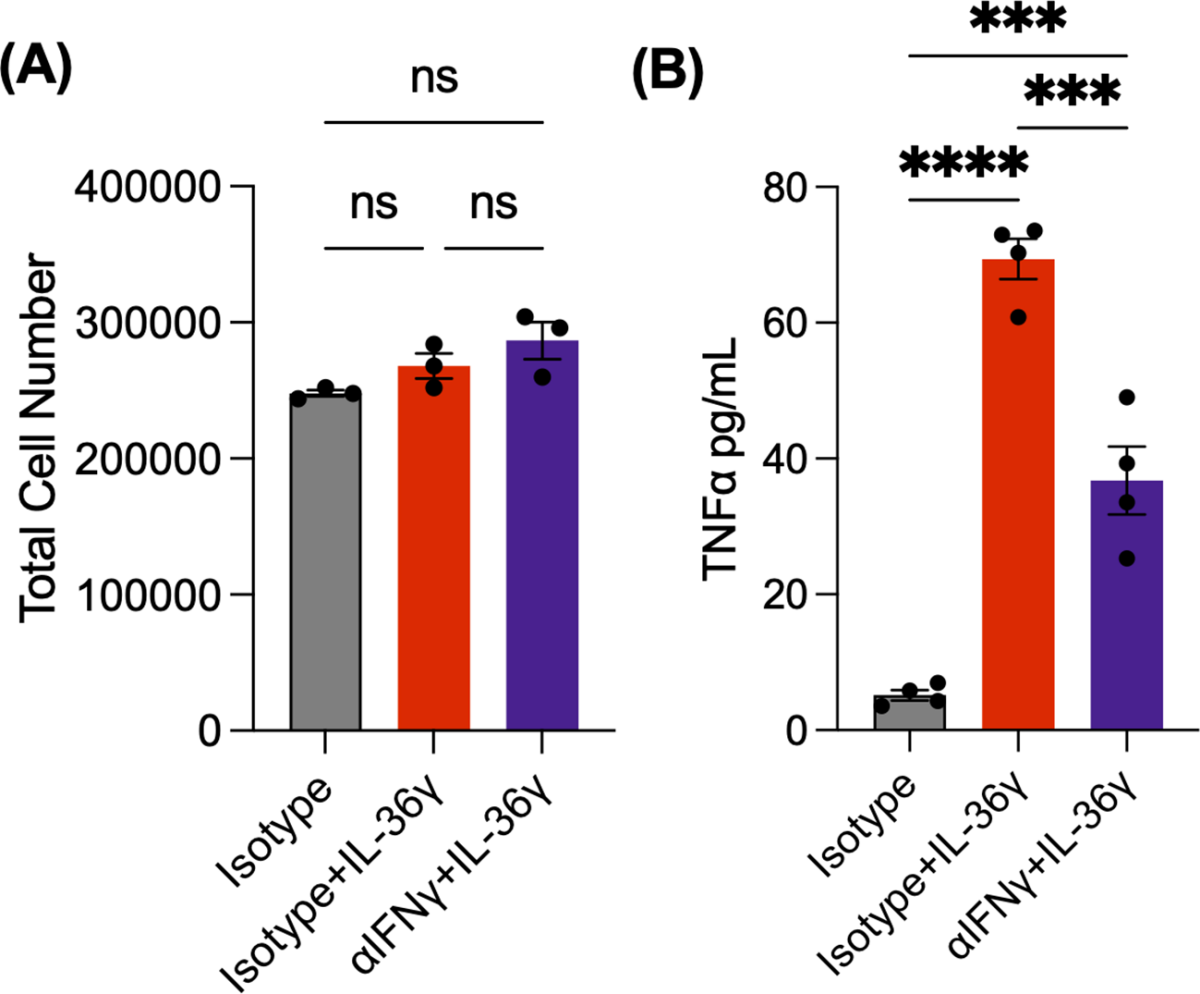
IL-36g-induced TNF*α* production by naïve CD4^+^ T cells is IFNγ dependent. **(A)** Cell number of naive CD4+ T cells stimulated with αCD3/αCD28 +IL-36γ, either with αIFNγ or an isotype antibody at 48 hours. As a control, some cells were stimulated but only received the isotype. **(B)** TNFα concentrations evaluated by ELISA. All data are presented as mean ± SEM; ***p < 0.001; ****p <0.0001; n.s., not significant, one-way ANOVA with Tukey’s multiple comparison test.

To test the link between IL-36γ and IFNγ, we carried out a similar *in vitro* experiment using naive CD4^+^ T cells and an IFNγ neutralizing antibody (αIFNγ). The cells were stimulated with αCD3/αCD28 and IL-36γ in the presence of αIFNγ or an isotype control antibody. As an additional control, some cells were stimulated without IL-36γ but received the isotype control antibody. After 48 hours, the cells were counted. We observed a higher cell number in IL-36γ stimulated samples, compared to the samples that only received the isotype control antibody (**Figure 3A**). TNFα was also measured via ELISA. While IL-36γ induced TNFα production by naive CD4^+^ T cells, neutralizing IFNγ reduced its secretion (**Figure 3B**). This suggests that IFNγ is a key intermediary in IL-36γ-induced TNFα production in CD4^+^ T cells.

### IL-36g induces robust colitis via the secretion of IFN*γ* by CD4^+^ T cells

**Figure 4:**
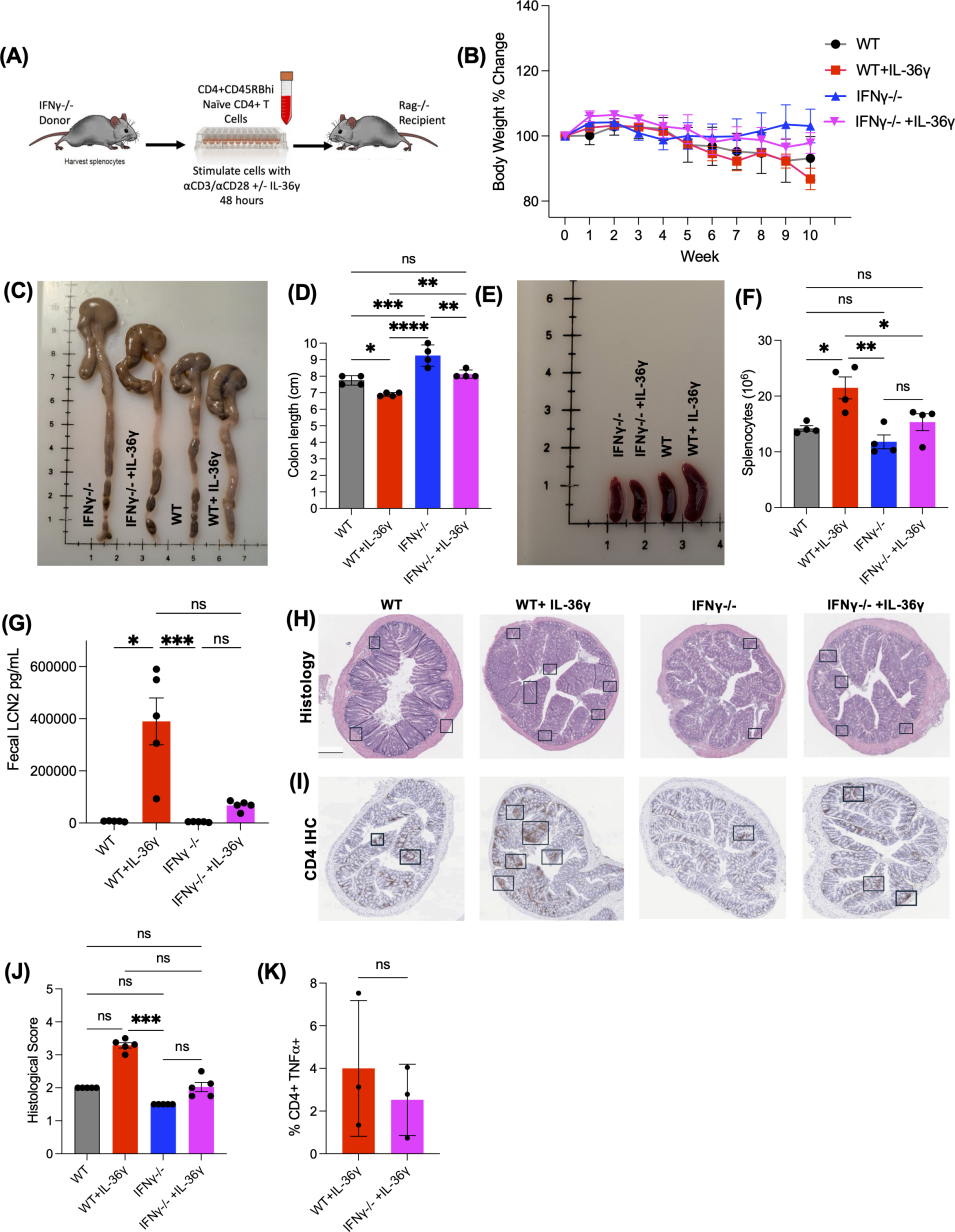
IL-36g induces robust colitis via the secretion of IFN*γ* by CD4^+^ T cells. **(A)** Diagram showing the adoptive transfer model using donor IFNγ-/- mice as donors and Rag-/- recipients. Naive CD4+ T cells are isolated and stimulated *in vitro* in the presence or absence of IL-36γ for 48 hours prior to the transfer. **(B)** Weight of the recipient mice was monitored during the time of the experiment over the course of 10 weeks. **(C)** Image of the harvested colons show that mice that received cells stimulated with IL-36γ cause the most inflammation to the colon, making it the shortest in length **(D)**. **(E, F)** Images of the harvested spleen show an enlarged spleen for the WT+IL-36γ group, with elevated splenocyte count. **(G)** Levels of fecal LCN2 levels were evaluated at time of euthanasia by ELISA. **(H)** H&E staining of colon sections from mice that received WT or IFNγ-/- naïve CD4+ T cells, in the presence or absence of IL-36γ. **(I)** Immunohistochemistry staining for CD4, of the same colonic tissue samples. **(J)** Histology scoring of colon sections from the same mice. **(K)** After euthanasia and tissue collection, TNFα production in the mesenteric lymph nodes was evaluated by ICCS. All data are presented as mean ± SEM; *p < 0.05, **p < 0.01, ***p < 0.001; ****p <0.0001; n.s., not significant, one-way ANOVA with Tukey’s multiple comparison test.

Next, we examined the influence of IL-36γ on IFNγ-dependent intestinal inflammation by transferring IL-36γ-stimulated IFNγ^-/-^ naïve CD4^+^ T cells into Rag^-/-^ mice. Prior to the transfer, naïve WT or IFNγ^-/-^ CD4^+^ T cells were stimulated with αCD3/αCD28 *in vitro* in the presence or absence of IL-36γ (**Figure 4A**). The mice were monitored over 10 weeks for their activity, weight, stool consistency, and LCN2 levels. Over time, mice transferred with WT cells +/- IL-36γ became less active and physically weaker. On the other hand, the mice that received IFNγ^-/-^ cells, in the presence or absence of IL-36γ, remained active. Mice that received WT + IL-36γ cells lost the most weight, followed by the WT group (**Figure 4B**). On the other hand, mice that received IL-36γ-stimulated IFNγ^-/-^ cells had minimal weight loss similar to recipients of IFNγ^-/-^ cells (**Figure 4B**). Ten weeks post transfer, mice were euthanized, and their colons and spleens were harvested for further analysis. Colons isolated from recipients of WT CD4^+^ T cells were noticeably shorter in length, especially WT + IL-36γ, when compared to the IFNγ^-/-^ recipients (**Figures 4C, D**). Accompanying the shorter colons was splenomegaly (**Figure 4E**), which is also reflected in splenocyte number (**Figure 4F**). Stool samples were checked for LCN2 levels. Consistent with the weight loss data, the WT + IL-36γ group had the highest LCN2 concentrations (**Figure 4G**). Unexpectedly, recipients of IL-36γ-stimulated IFNγ^-/-^ cells exhibited elevated levels of LCN2 even though they had minimal weight loss (**Figures 4B, G**). The other two groups, recipients of WT and IFNγ^-/-^ CD4^+^ T cells, had low levels of fecal LCN2 (**Figure 4G**). H&E staining further detailed a thickening of the submucosa and leukocyte infiltration predominantly in the WT + IL-36γ colons when compared to the other groups (**Figure 4H**). The same colon tissue was stained for CD4 using immunohistochemistry, where both groups (WT + IL-36γ and IFNγ^-/-^ + IL-36γ) had the most CD4 infiltration (**Figure 4I**). Overall, colons isolated from WT + IL-36γ had the highest histological scores (**Figure 4J**). Corroborating this, when IFNγ^-/-^ naïve CD4^+^ T cells were transferred to Rag^-/-^ mice to assess intestinal inflammation, we observed a reduction in TNFα levels in mesenteric lymph nodes using ICCS (**Figure 4K**).

## Discussion

IL-36 signaling is known for promoting inflammatory signaling in the skin, lungs, kidneys, and intestinal inflammation during IBD and experimental colitis. In IBD, IL-36 ligands (IL-36α, IL-36β, and IL-36γ) are expressed by gut lymphocytes, macrophages, and intestinal epithelial cells. The activation of the IL-36 receptor induces NF-kB and MAPK in a MYD88 dependent manner, which induces other pro-inflammatory pathways by target cells in the colon.

When it comes to IL-36γ signaling, some studies show a pathogenic role, while other studies found that IL-36γ can aid in healing of the disease. A previous study from our lab suggested a protective role for IL-36γ in the DSS colitis model by helping in barrier restoration (28); however, using an oxazolone model, a pathogenic role for the cytokine has also been observed (29). The discrepancy in these data may be due to differences in the chemical used to induce colitis. Moreover, as chemical agents are used to promote colitis in the DSS and oxazolone model systems, it is unclear whether the observed role of IL-36γ in each system is specific to the model or a “double-edged sword” role of IL-36 g in colitis in general. Since IBD is heavily mediated by T cell responses, and IL-36 cytokines are known to promote the recruitment of T cells (30) and induce the secretion of other pro-inflammatory cytokines (31), we investigated the influence of IL-36γ on naïve CD4^+^ T cells and colitis using a well-known adoptive T cell transfer model of colitis (10).

Our results demonstrate that stimulating naïve CD4^+^ T cells with IL-36γ significantly induced cell expansion *in vitro* and colitis *in vivo*. This could be due to IL-36γ providing survival signals, enhancing proliferation, or both. While deciphering between these scenarios is outside the scope of the current study, the data demonstrating no difference in cell death (data not shown) suggest that IL-36γ works by enhancing the proliferative capacity of CD4^+^ T cells by increases CD4^+^ T cell numbers. Accompanying IL-36γ stimulation of CD4^+^ T cells was notable expression and production of the Th1 pro-inflammatory cytokine IFNγ by naïve CD4^+^ T cells compared with the other IL-36 family members (**Figure 1**). This was expected, as previous studies showed IL-36 cytokines induce the production of IFNγ (32, 33). This IL-36γ -induced IFNγ was responsible, at least in part, for the ability of IL-36γ to drive robust colitis induction. Despite the previously reported studies, which highlight the highest IFNγ production by naïve CD4^+^ T cells when stimulated with IL-36β (30), T cell exhaustion may have occurred due to higher concentrations of the costimulatory molecules, αCD3/αCD28 (34) in addition to IL-36β which caused a decrease in IFNγ production. Further, IFNγ was identified as an upstream mediator of IL-36γ-induced TNFα production *in vitro*, and potentially *in vivo*.

We evaluated the relationship between IL-36 cytokines and IFNγ by neutralizing IFNγ *in vitro* in the presence of these cytokines. Our data suggests that blocking IFNγ can suppress the expression and secretion of the pro-inflammatory cytokine TNFα. When naïve CD4^+^ T cells were stimulated with IL-36γ and transferred into Rag^-/-^ mice, the mice lost weight, developed loose stool consistency over time, and had the highest fecal LCN2 levels. The mice in this group also became visibly weaker over time. They appeared to be less motivated to explore and engage in social interactions. The colon was also notably shorter in length and had thicker tissue. IFNγ^-/-^ naïve CD4^+^ T cells were also stimulated with IL-36γ and transferred into Rag^-/-^ mice. Despite their elevated fecal LCN2 levels, weight loss was not significantly different in mice that received IFNγ-/- naïve CD4^+^ T cells stimulated with IL-36γ. However, they did not lose a significant amount of weight. Their colons were longer, and they had mild levels of fecal LCN2. This suggests that IL-36γ drives IFNγ-dependent T cell mediated colitis.

Based on the available pre-clinical studies on pro-inflammatory cytokines in the T cell mediated colitis model, understanding the role of IL-36γ in intestinal inflammation will highlight new therapeutic approaches. While we employed a model for *in vitro* stimulation of CD4^+^ T cells with IL-36γ, it is likely that during intestinal inflammation, IL-36γ is induced by microbiota-dependent stimulation of macrophages or other innate immune cells following barrier disruption. In turn, this leads to CD4^+^ T cell activation and differentiation into Th1 and/or pathogenic T cells that produce IFNγ and other pro-inflammatory cytokines.

Similar to the findings by Powrie et al., we also found that the role of IFNγ is significant in developing intestinal inflammation and colitis, especially in the adoptive transfer model. However, we acknowledge that our adoptive transfer of freshly isolated naive CD4^+^ T cells demonstrates milder disease severity than reported in the literature. Outside the scope of the current study, we predict the observed discrepancy in disease severity despite using a similar model system likely due to environmental differences. It is well established that the trajectory and severity of colitis in mice is dependent on the facility in each the mice are housed. Moreover, freshly isolated naive CD4^+^ T cells were mock-stimulated *in vitro* prior to transfer to control for the *in vitro* conditions mentioned. Nevertheless, our study presents novelty in the adoptive transfer model described here, highlighted through the stimulation of naïve CD4+ T cells *in vitro* before transferring them to the immunocompromised mice.

In conclusion, our results demonstrate a potential for IL-36γ and IFNγ to be mutually targeted as therapeutics to alleviate colitis symptoms and perhaps prevent IBD, in the long run. Most of the available research targets downstream drivers of IBD, which narrows down the focus to specific molecules or pathways. Such downstream targets include TNFα, the IL-12/IL-23 axis, IL-23/IL-17 axis, JAK inhibitors, integrins and receptor modulators (35). Some antibodies and biological therapies were developed to interfere with the function of these molecules and pathways, such as Anti-TNF therapy, anti-IL-12/23, anti-integrins, and JAK inhibitors- which have been approved for treating UC or CD. Although this wide variety of therapeutics has improved some patients’ lives, they are not universally effective. Moreover, many patients lose responsiveness to therapy over time (36). Previous studies investigated the impact of blocking IL-36 signaling by administering neutralizing monoclonal antibodies or the receptor antagonist IL-36Ra. A humanized monoclonal antibody developed to block IL-36R signaling called Spesolimab, was used in patients with moderate to severe UC. It was well-tolerated by UC patients, although, the efficacy endpoints were not reached, despite it being FDA approved for general pustular psoriasis flares. Alternatively, the administration of IL-36Ra to inhibit the IL-36R signaling pathway in a colon cancer mouse model showed a reduction in tumor cell proliferation. This is consistent with a previous *in vivo* study that showed increased incidence of colon tumorigenesis in the absence of IL-36Ra (37). These results suggest that targeting upstream drivers, such as IL-36 cytokines, may have better therapeutic effects during intestinal inflammation, as it would target downstream cytokines, such as TNFα and IFNγ.

## Data Availability

The original contributions presented in the study are included in the article/Supplementary Material. Further inquiries can be directed to the corresponding author.
